# The Prevalence, Coexistence, and Correlations between Seven Pathogens Detected by a PCR Method from South-Western Poland Dairy Cattle Suffering from Bovine Respiratory Disease

**DOI:** 10.3390/microorganisms10081487

**Published:** 2022-07-24

**Authors:** Agnieszka Lachowicz-Wolak, Małgorzata D. Klimowicz-Bodys, Katarzyna Płoneczka-Janeczko, Marek Bykowy, Magdalena Siedlecka, Jagoda Cinciała, Krzysztof Rypuła

**Affiliations:** 1 Division of Infectious Diseases of Animals and Veterinary Administration, Department of Epizootiology and Clinic of Birds and Exotic Animals, Faculty of Veterinary Medicine, Wroclaw University of Environmental and Life Sciences, pl. Grunwaldzki 45, 50-366 Wroclaw, Poland; agnieszka.lachowicz-wolak@upwr.edu.pl (A.L.-W.); malgorzata.klimowicz-bodys@upwr.edu.pl (M.D.K.-B.); katarzyna.ploneczka-janeczko@upwr.edu.pl (K.P.-J.); marek.bykowy@upwr.edu.pl (M.B.); magdalena.siedlecka@upwr.edu.pl (M.S.); 2 Student Scientific Society “AnthraX”, Department of Epizootiology and Clinic of Birds and Exotic Animals, Faculty of Veterinary Medicine, Wroclaw University of Environmental and Life Sciences, pl. Grunwaldzki 45, 50-366 Wroclaw, Poland; 115803@student.upwr.edu.pl

**Keywords:** BRD, pathogen configurations, pathogen correlations, coinfections, PCR detection, cattle, prevalence, respiratory diseases

## Abstract

Bovine respiratory disease (BRD) is a very important disease that contributes to economic losses in dairy and beef cattle breeding worldwide. The molecular testing of material from 296 calves showing BRD symptoms from 74 dairy herds located in south-western Poland was performed in 2019–2021. Molecular tests were performed using a commercial kit “VetMAX^TM^ Ruminant Respiratory Screening Kit” (Thermo Fisher Scientific) for the simultaneous detection of genetic material of seven pathogens responsible for BRD. At least one pathogen was detected in 95.95% of herds. The overall prevalence was: *Pasteurella multocida* 87.84%, *Mannheimia haemolytica* 44.59%, bovine coronavirus (BcoV) 32.43%, *Mycoplasma bovis* 29.73%, *Histophilus somni* 28.38%, bovine parainfluenza virus type 3 (BPIV-3) 13.51%, and bovine respiratory syncytial virus (BRSV) 10.81%. Twenty-nine configurations of pathogen occurrences were found. Bacterial infections were the most frequently recorded as 56.7% of all results. Coinfections mainly consisted of two pathogens. Not a single purely viral coinfection was detected. The most frequent result was a single *P. multocida* infection accounting for 18.31% of all results. The statistically significant correlation (*p* = 0.001) with the highest strength of effect (ϕ 0.38) was between *M. bovis* and *H. somni*.

## 1. Introduction

Bovine respiratory disease (BRD) is one of the most significant causes of economic losses in dairy and beef cattle breeding worldwide. BRD is a polyetiological disease with viral and bacterial causes, to which environmental conditions and stress may contribute [1,2]. The disease is most prevalent in the fall and spring months [3,4,5,6]. Young animals, less than a year old, more commonly suffer from this [7,8,9,10,11].

Pathogens noted in the course of BRD include: *Pasteurella multocida* (*P. multocida*), *Mannheimia haemolytica* (*M. haemolytica*), *Mycoplasma bovis* (*M. bovis*), *Histophilus somni* (H. somni), bovine parainfluenza virus type 3 (BPIV-3), bovine respiratory syncytial virus (BRSV), bovine coronavirus (BcoV), bovine viral diarrhea virus (BVDV), bovine herpes virus type 1 (BHV-1), bovine adenovirus (BadV), and influenza D virus (IDV). Stress or viral infections create a basis for opportunistic bacteria to multiply, which contributes to clinical symptoms [2,5,7,8,9,10,12,13,14,15,16,17,18,19,20,21].

Symptoms include nasal and ocular discharge of serous, serous-mucous, and purulent fluid, fever, cough, and increased respiratory rate [7,8,9,10,12,13,22,23]. Heifers affected by BRD reach first mating weight later than those not affected and pneumonia delays first calving by half a month [22,24]. In addition, pneumonia contributes to a lower first lactation performance [22,24,25].

Data show that in the U.S., losses associated with BRD in meat calves after weaning were approximately USD 165 million per year, of which costs associated with falls were USD 126 million, those associated with treatment were USD 25 million, and those associated with reduced calf weight after BRD were USD 15 million [26].

The prevalence of individual infectious agents is at highly variable levels in different scientific reports. Moreover, so far, there are no studies on the simultaneous occurrence of factors responsible for BRD among Polish cattle. Determining the prevalence of individual pathogens and the correlation between them can help to develop more effective management and prevention strategies for BRD in this geographic area. It may also contribute to the understanding of the interrelationships between pathogens and to the better knowledge of calves’ nasal bacterial communities [27,28,29]. The aim of this study was the epidemiology of BRD along with the determination of the prevalence and relationships between seven pathogens detected in the course of BRD in sick dairy calves from south-western Poland to improve the knowledge on coinfections in BRD.

## 2. Materials and Methods

### 2.1. Sample Collection

**Figure 1:**
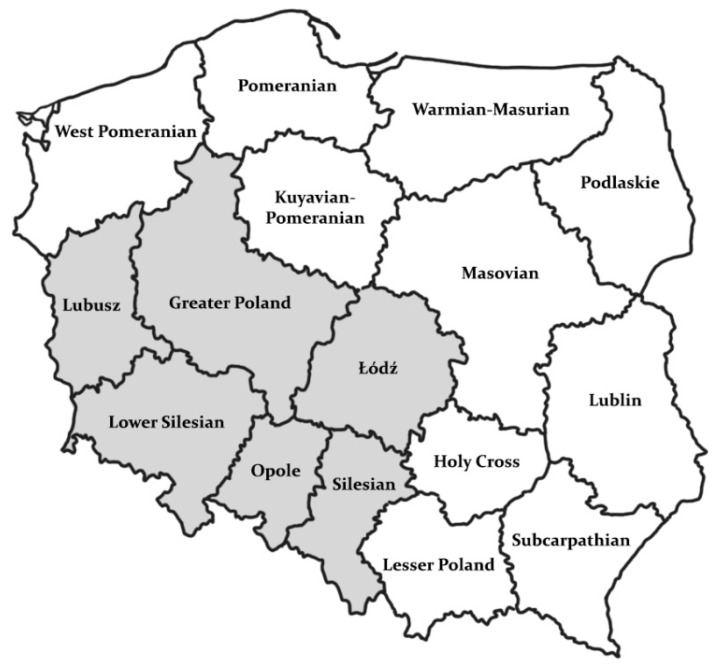
Map of Poland with region marked in gray where tested dairy cattle herds were located.

The study was conducted in the period from January 2019 to January 2021. The material came from dairy cattle herds located in south-western Poland from the following voivodeships: Greater Poland, Lodz, Lower Silesian, Lubusz, Opole, Silesian (Figure 1).

In the study region, dairy herds are kept in two types of farms. Free-stall, often with free access to paddocks, and tethered. The free-stall type prevails. Animals are usually kept on bedding of varying depths. In a very small number of farms, cattle have access to pasture [30]. Based on preliminary studies (unpublished data), *P. multocida* was considered the leading pathogen (prevalence 75%). In addition, literature data from Belgium, which has a similar weather to Poland and is at a similar latitude, gave a similar prevalence (73.3%) [19].

The number of farms where animals should be examined was determined based on the following formula [31] (at a confidence level of 0.95) and was 74.

n = [1.962 × P_exp_ × (1 − P_exp_)]/d^2^ (1)

n—required sample size;
P_exp_—expected prevalence (0.74);
d—desired absolute precision (0.1).

The number of samples per herd was determined based on the formula [31] (at a confidence level of 0.95):

n = [1 − (1 − p_1_)^1/d^] × [N − (d/2)] + 1 (2)

n—number of samples submitted from the herd;
p_1_—probability to detect at least one infected calf (0.95);
N—number of animals in the herd;
d—minimum number of affected animals expected in the herd (N × expected prevalence).

Taking into account the herd size (number of animals per farm) and the prevalence used to determine the number of farms, the calculated number of samples per herd was 4.

According to the above calculations, material was collected from 4 calves from each herd. Samples were pooled by combining the collected material to obtain one representative test result for material from animals from a single herd.

Material was collected in cooperation with veterinarians dealing with tested herds. Samples were collected by veterinarians from calves not previously vaccinated against the tested infectious agents. Tested calves showed symptoms of BRD such as nasal and ocular discharge, fever, cough, increased respiratory rate, increased lung field murmur, respiratory wheezing, and rales. The material consisted of deep nasal swabs (NS) or tracheal washes (TW) from calves showing signs of respiratory infection. Material was collected with due aseptic care and attention to avoid contamination.

### 2.2. PCR Test

Collected samples were transported within 24 h to the laboratory. The study was performed at the Wroclaw University of Environmental and Life Sciences, in the laboratory of the Department of Epizootiology and Clinic of Birds and Exotic Animals. From the obtained samples, the genetic material of the tested pathogens was isolated using a commercial kit “Total RNA Mini Plus” (A&A Biotechnology, Gdynia, Poland) following the manufacturer’s instructions at each step. The RNA-DNA was amplified in a CFX96 Connect Real-Time PCR Thermal Cycler (Bio-Rad, Marnes-la-Coquette, France). Molecular tests were performed using a commercial kit “VetMAX^TM^ Ruminant Respiratory Screening Kit” (Thermo Fisher Scientific, Lissieu, France) simultaneously detecting seven bovine respiratory pathogens, following the manufacturer’s instructions at each step. This test detects: *Pasteurella multocida* (*P. multocida*), *Mannheimia haemolytica* (*M. haemolytica*), *Histophilus somni* (*H. somni*), *Mycoplasma bovis* (*M. bovis*), bovine respiratory syncytial virus (BRSV), bovine coronavirus (BCoV), and bovine parainfluenza virus type 3 (BPIV-3).

### 2.3. Statistical Analysis

The analyzed unit of this study was the herd. The “prevalence” means the percentage of affected herds by the analyzed pathogen.

Cross-tab analyses were performed along with chi-square tests (χ^2^ test) to check for correlations between the cooccurrence of the pathogens studied. In addition, it was checked whether there was a relationship between the occurrence of viruses and bacteria. The strength of the effect of the tested compounds was determined using the phi Yule contingency coefficient (ϕ) for which the cut-off values were:

ϕ < 0.1—no effect;

0.1 < ϕ ≤ 0.3—small effect;

0.3 < ϕ ≤ 0.5—average effect;

0.5 < ϕ—large effect.

The Phi Yule contingency coefficient is considered statistically significant if the *p* value is equal to or less than the significance level α. The significance level in this chapter was considered to be α = 0.05.

Combinations without repetitions of occurrences of individual pathogens in this article are also called “configurations”, and the number of possible combinations was calculated by the binomial coefficient. For comparing the similarity and diversity of coexistence, we used a Jaccard similarity coefficient (Jaccard index). Statistical analyses were performed using Statistica 13.3.721.1 (TIBCO Software Inc., Palo Alto, CA, USA). Tables and graphs were prepared using Microsoft Office Excel. Euler diagrams were prepared using Adobe Illustrator.

### 2.4. Ethics Statement

Ethical review and approval were waived for this study due to the present law in Poland (the Experiments on Animals Act from 15 January 2015, Journal of Laws of the Republic of Poland from 2015, item. 266): the study did not require the approval of the Ethics Committee. The samples used in this study originally came from the material for a diagnostic of cattle infections that was collected by veterinarians treating these herds. Additionally, it did not cause the animals any pain, suffering, or distress equal to or greater than a needle stick injury. However, the research outline was submitted to the Animal Welfare Advisory Team in Wroclaw, which qualified the study as research that did not require ethics committee approval.

## 3. Results

**Figure 2:**
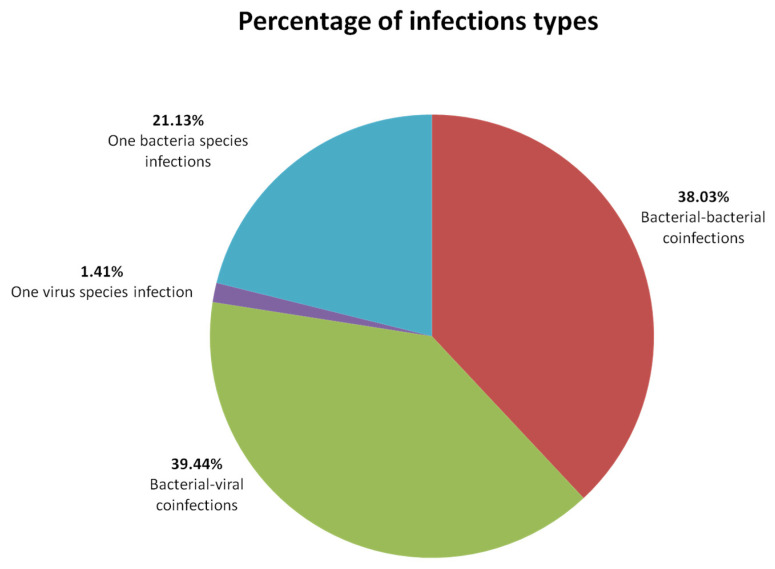
Percentage of infection types in positive results. Virus–virus coinfections were not detected.

A total of 296 calves showing BRD symptoms from 74 herds located in south-western Poland were examined. In 27 herds, the test material was tracheal washes, and in 47 herds, the test material was nasal swabs. The tests showed the presence of genetic material of at least one of the pathogens in 71/74 (95.95%) of the examined herds. In herds where the test material was tracheal washes, positive herds were 26/27, and when the test material was nasal swabs, positive herds were 45/47. Bacterial infections were the most frequently recorded 42/74 (56.7%). The structure of infection types from positive results (71) is shown in the pie chart (Figure 2).

### 3.1. Prevalence

#### 3.1.1. Overall Prevalence

**Figure 3:**
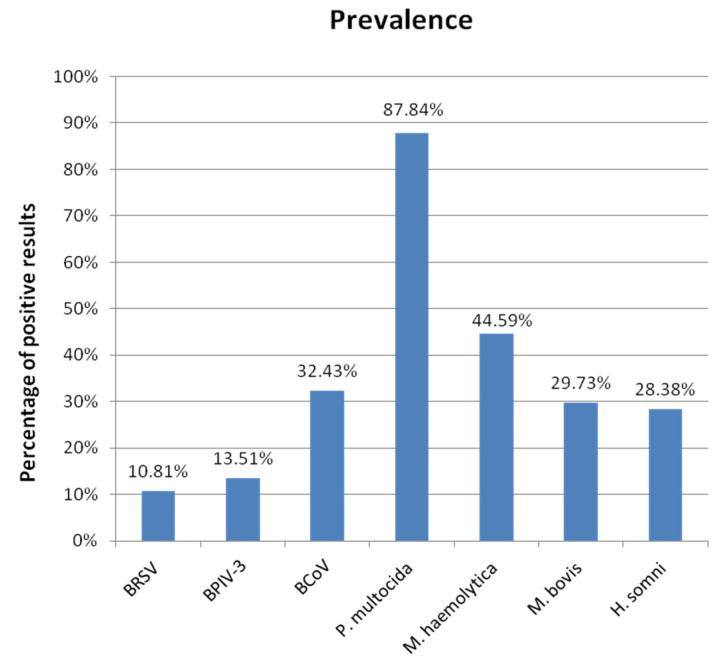
The overall prevalence of individual pathogens detected.

The overall prevalence of individual pathogens is shown in bar graph (Figure 3).

#### 3.1.2. Prevalence Depending on the Type of Samples

**Figure 4:**
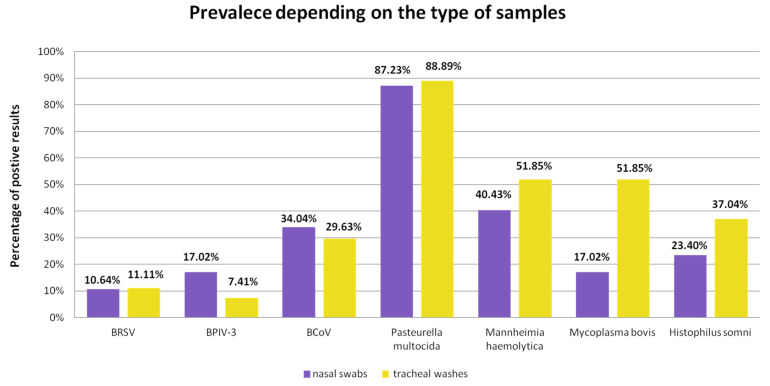
The prevalence of individual pathogens detected depending on the type of sample.

The prevalence of individual pathogens depending on the type of samples is shown in the bar graph (Figure 4).

### 3.2. Occurrences

Analyzing the results of the study according to the number of simultaneous detection of pathogens in each study, it was found that coinfections with two pathogens were most frequently recorded (22/74). Infections with three pathogens were found in 17 of the tested herds. One pathogen was found in 16 herds. Four pathogens were detected in 10 of the tested herds, five pathogens were found in 5 of the tested herds, and seven pathogens were detected in only one tested herd. Infection with six pathogens was not found in any of the tested herds. In three herds, none of the pathogens were detected.

### 3.3. Correlations

The statistical tests performed from all the obtained results did not confirm the existence of an association between the presence of viral and bacterial pathogens. Statistical tests performed showed five statistically significant associations between pairs of pathogens with an average and small effect (Table 1). The highest strength of effect was the correlation between *M. bovis* and *H. somni* ϕ 0.38, and its effect was defined as average. The next with an average effect was between BRSV and BPIV-3 ϕ 0.33, and between BCoV and BPIV-3. All statistically significant correlations shown meant that the pathogen under study was significantly more prevalent with the simultaneous occurrence of the other pathogen from the pair under study.

### 3.4. Configurations

#### 3.4.1. Occurred Configurations

In the results obtained, 29 configurations of pathogen occurrences were found. Results of individual PCR tests are presented in Table 2.

#### 3.4.2. Configurations in Bacterial-Only Infections and Coinfections

**Figure 5:**
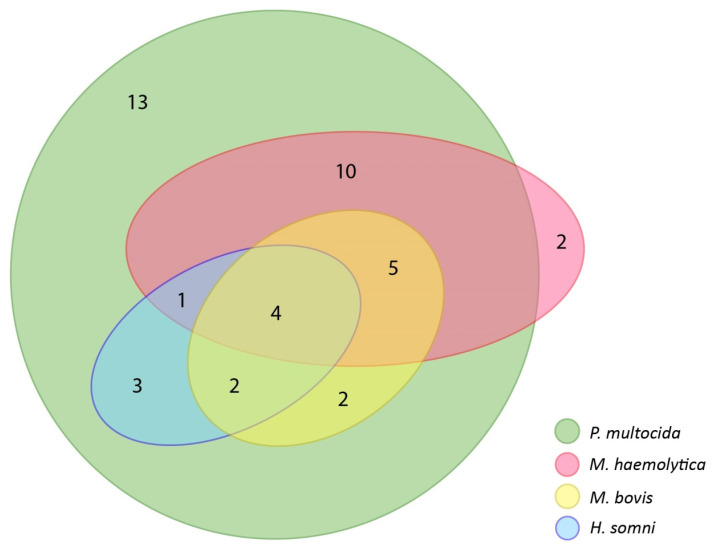
Euler diagram with numbers of occurring bacterial infections and bacterial-only coinfections. Common parts indicate the numbers of coinfections in which a common occurrence of pathogens shown was recorded. *H. somni* and *M. bovis* did not occur as a single pathogen infection.

Bacterial-only coinfections were reported in 27 herds, representing 38.03% of herds in which at least one pathogen was detected (27/71). The numbers of bacterial infections occurring and bacterial-only coinfections are shown using sets in Figure 5.

#### 3.4.3. Coexistence

**Figure 6:**
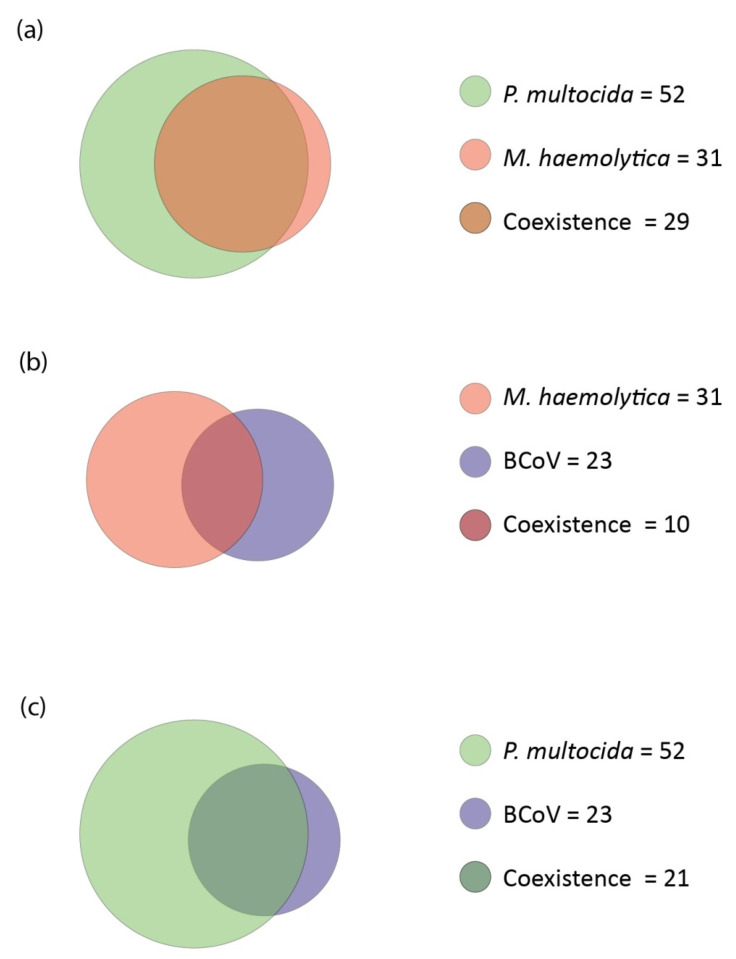
Area-proportional Euler diagrams with coexistence of the three pairs of pathogens with the highest recorded prevalence. The area of the circle corresponds to the number of occurrences of a given pathogen in coinfections. The common part indicates the number of coinfections in which the co-occurrence of both pathogens shown was recorded, regardless of the rest of the pathogens in the coinfection. (**a**) Coexistence of *P. multocida* and *M. haemolytica*. (**b**) Coexistence of *M. haemolytica* and BCoV. (**c**) Coexistence of *P. multocida* and BCoV.

The coexistence of the three pathogens showing the highest prevalence (Figure 6).

*M. haemolytica* with *P. multocida*

Coinfection of *M. haemolytica* with *P. multocida* accounted for 93.54% (29/31) of bacterial and viral-bacterial coinfections containing *M. haemolytica* regardless of the species of the other noted pathogens in a given configuration. For *P. multocida*, coexistent coinfection with *M. haemolytica* accounted for 55.77% (29/52) of the recorded coinfections.

*M. haemolytica* with BCoV

Coinfection of *M. haemolytica* with BCoV accounted for 32.56% (10/31) of bacterial and viral-bacterial coinfections containing BCoV regardless of the species of the other noted pathogens in a given configuration. For *M. haemolytica*, coexistent coinfection with BCoV accounted for 43.48% (10/23) of the recorded coinfections.

*P. multocida* with BCoV

Coinfection of *P. multocida* with BCoV accounted for 40.38% (21/52) of bacterial and viral-bacterial coinfections containing *P. multocida* regardless of the species of the other noted pathogens in a given configuration. For BCoV, coexistent coinfection with *P. multocida* accounted for 91.30% (21/23) of the recorded coinfections.

**Figure 7:**
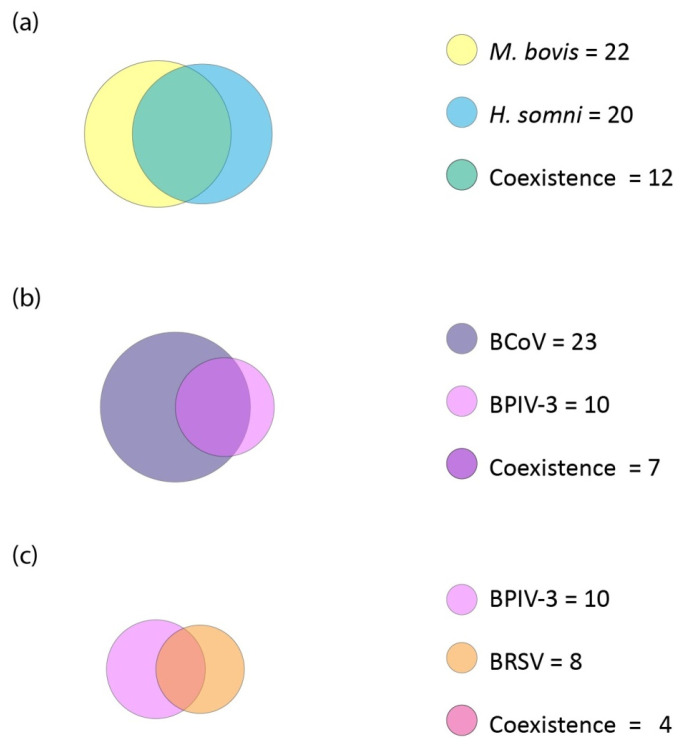
Area-proportional Euler diagrams with coexistence of the three pairs of pathogens for which the highest statistically significant correlation coefficients were shown overall. The area of the circle corresponds to the number of occurrences of a given pathogen in coinfections. The common part indicates the number of coinfections in which a common occurrence of both pathogens shown was recorded, regardless of the rest of the pathogens in the coinfection. (**a**) Coexistence of *M. bovis* and *H. somni*. (**b**) Coexistence of BCoV and BPIV-3. (**c**) Coexistence of BPIV-3 and BRSV.

The coexistence of the chosen three pairs of pathogens with statistically significant correlations for which the highest strength of association effect was shown (Figure 7).

*M. bovis* with *H. somni*

Coinfection of *M. bovis* with *H. somni* accounted for 54.55% (12/22) of bacterial and viral-bacterial coinfections containing *M. bovis* regardless of the species of the other noted pathogens in a given configuration. For *H. somni*, coexistent coinfection with *M. bovis* accounted for 60.00% (12/20) of the recorded coinfections.

BCoV with BPIV-3

Coinfection of BCoV with BPIV-3 accounted for 30.43% (7/23) of viral-bacterial coinfections containing BCoV regardless of the species of the other noted pathogens in a given configuration. For BPIV-3, coexistent coinfection with BCoV accounted for 70.00% (7/10) of the recorded coinfections.

BRSV with BPIV-3

Coinfection of BRSV with BPIV-3 accounted for 50.00% (4/8) of viral-bacterial coinfections containing BRSV regardless of the species of other noted pathogens in a given configuration. For BPIV-3, coexistent coinfection with BRSV accounted for 40.00% (4/10) of the recorded coinfections.

**Figure 8:**
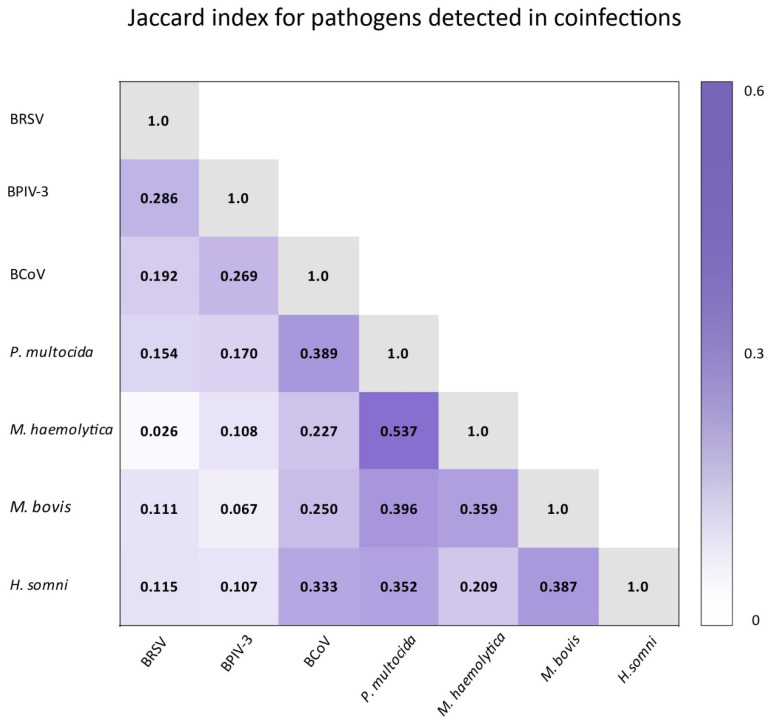
Matrix of Jaccard similarity coefficients (Jaccard index) between all pathogens detected in coinfections. The darker the color, the higher the value of the Jaccard index.

Jaccard index for all pathogens detected in coinfections (Figure 8).

## 4. Discussion

To the authors’ knowledge, no study on the simultaneous occurrence of the few pathogens in the course of BRD in calves has been published from Polish herds to date. In addition, the comparison of data on coinfections, especially the configuration of infectious agents responsible for BRD, is not easy, because little literature data are available and are due to the multifactorial nature of this disease entity, in which at least a dozen pathogens are involved. As a result, virtually all published studies of this type detected a different set of pathogens. In this study, the commercial kit “VetMAX^TM^ Ruminant Respiratory Screening Kit” (Thermo Fisher Scientific, Lissieu, France) was used, which will allow other researchers to compare data in the future. A test from the same product line, i.e., VetMAX^TM^, detecting the same pathogens was used by Paller et al. who also examined calves showing BRD symptoms from Slovenia [32]. They showed that 82.7% of the samples tested contained at least one test factor [32], and in our study, the percentage of positives was also very high at 95.95%.

### 4.1. Infection Types

According to the tests, mainly bacterial infections were recorded. In this study, the only case of purely viral infection was the occurrence of BCoV. Coinfections with two pathogens were most frequently recorded (22/74). Although Paller et al. additionally tested samples for three viruses other than ours (BVDV, BAdV, and BHV-1), reporting results for a total of 10 pathogens, the most commonly reported result, regardless of pathogen type, was coinfection with two pathogens [32], similar to the results of our study. Oliveira et al. who tested cattle in Brazil showed in more than 1/3 of the studied samples the presence of genetic material of only one agent [33], while in our study, mainly coinfections were recorded. This may be due to the fact that our study is a field study, commissioned by farm doctors. As a PCR test that simultaneously detects seven pathogens is often ordered in very problematic or chronic cases, bacterial complications may be more frequent [24,34].

### 4.2. Prevalence

The most frequent pathogens among dairy herds in south-western Poland are *P. multocida* (87.84%) and *M. haemolytica* (44.59%). The most frequent viral pathogen was BCoV (32.43%). The prevalence of BRD individual infectious agents is at highly variable levels in different scientific reports and it could be even different in other parts of the same country.

The prevalence of *P. multocida* varies widely ranging from a few percent to over eighty [5,8,14,15,16,17,19,20,28,32,35,36,37,38]. Similar to our results, Paller et al. noted that the highest prevalence occurred for *P. multocida* (58.64%) [32]. Pratelli et al. tested calves for the same pathogens as we did in our study and additionally for bovine adenovirus. Calves showing BRD symptoms had the highest prevalence of *H. somni* (84.8%), BCoV (74.6%), and *P. multocida* (50.0%) [8]. In our study, BCoV was also one of the three most frequently recorded pathogens, although its prevalence was not as high, just as we did not record as high a prevalence of H.somni [8,9,28,38]. The prevalence of these pathogens also varies depending on the publication, although BCoV is often reported at levels in the tens of percent [6,8,9,20,39,40,41,42].

BPIV-3 prevalence in the majority of publications is low, ranging from a few to several percent [6,7,8,32,33,35], even if sometimes recorded at a higher level, and BPIV-3 was also not considered a leading pathogen [17]. The situation is different for the prevalence of BRSV, which, in some studies, is presented as the most important viral agent with a prevalence of about 40% [17,32,39]. In our study, BRSV was the least reported of all pathogens, with a prevalence of 10.81%, so it was more similar to the level shown by Klima et al. [35] and Pratelli et al. [8]. In some studies, its prevalence is even lower [7]. Research of Urban-Chmiel et al. showed that in another region of Poland (the east), BRSV prevalence among dairy cattle herds was 66.6% [43]. Thus, although BRSV is considered to be widespread worldwide, as can be seen, its prevalence varies from region to region. The situation is similar for the prevalence of *M. bovis*. A study conducted by Szacawa et al. in a part of eastern Poland showed a prevalence of *M. bovis* in calves of 56.5% [44], which is higher than the percentage shown by us (29.73%). However, their study was conducted on a smaller group, as well as a different region of Poland [44]. The prevalence of *M. bovis* shown by researchers from other regions of the world varies even more: from a few percent to over eighty [8,15,16,19,28,32,33,35].

We showed that for BPIV-3 and BCoV virus detection, samples were more often positive when the material was nasal swabs than tracheal washes. The opposite was true for bacterial detection, with a particularly large difference in percentage points seen in the prevalence of *M. bovis*. BRSV was also more frequently detected in herds where the test material was tracheal swabs, although this was a small difference of 0.47 percentage points. In our study, only one type of sample was collected from calves from a single herd, so we cannot compare which type of material is better for BRD diagnosis. Comparative studies have been conducted by other researchers [29,45,46,47,48,49]. There is no consensus on which samples are most suitable for BRD diagnosis [34,50]. Doyle et al. showed that to detect bacteria, which was the subject of our study, in the course of BRD, the site of collection of material from the respiratory tract will be of little importance, and for viruses, better material will be that from the upper respiratory tract, especially for the detection of BCoV [45]. Different results were presented by Zhang et al.—BRSV and BCoV were more frequently noted in tracheal washes [46]. On the other hand, some studies showed that *M. bovis* was more often noted when the material came from the lower respiratory tract [48,49]. With these results and animal welfare in mind, we share the view of Pardon et al.—samples from the upper respiratory tract might be sufficient and even most appropriate to detect primary pathogens [34]. Nevertheless, if the disease lasts longer, and bacteria are suspected as the main complicating factor, then the more appropriate material for testing will be that from the lower respiratory tract.

### 4.3. Coinfections and Configurations

In the results obtained, only 29 configurations of pathogen occurrences were found, despite the fact that the mathematically possible number of individual configurations is 127. Thus, with 74 herds tested, it was possible that no test result would be repeated, which, however, was not the case. The most common result was a single *P. multocida* infection (13/71) accounting for 18.31% of all positive results. No purely viral coinfections were detected. Of the 15 possible configurations of bacterial coinfections, only 7 were found. These 7 configurations that occurred are all possible configurations containing *P. multocida*. Paller et al. noted in their study that *P. multocida* genetic material was detected most frequently in samples where a single agent was recorded. However, it can be noted that in configurations containing two pathogens, those containing *P. multocida* were the most frequently recorded [32], similar to our results. Schönecker et al. who tested cattle in Switzerland in their study also reported coinfections and configurations, however, for a different set of pathogens. Similar to us, they showed that the most frequently detected agent in monovalent infections was *P. multocida*. However, the most common coinfection was *P. multocida* and *M. bovis* accounting for 6.2% of all results [37]. In our study, the highest number of bacterial coinfections were cases of the simultaneous presence of *P. multocida* and *M. haemolytica* (10/71), which constituted 14.08% of all positive results, but at the same time, it accounted for 37.04% (10/27) of all bacterial coinfections. However, when coinfections with only two pathogens are discarded, a high percentage (45.23%) of *M. haemolytica* is also seen in the remaining coinfections with *P. multocida*, regardless of the species of the rest of the pathogens. In other words, *M. haemolytica* is present in 45.23% of coinfections with three or more pathogens in which *P. multocida* is noted. Such infections (*P. multocida* and *M. haemolytica*) are twice as many as the next most common bacterial coinfection configuration: *P. multocida*, *M. haemolytica*, and *M. bovis* (5/71), which account for 18.52% (5/27) of bacterial coinfections. It is also noteworthy that this configuration is a replication of the most common coinfection, extended with *M. bovis*. On the other hand, the least frequently recorded bacterial coinfection is *M. haemolytica*, *P. multocida*, and *H. somni*. This bacterial configuration appears only once, accounting for only 3.70% (1/27) of bacterial coinfections, yet 1.41% (1/71) of all positive results. However, this bacterial configuration extended by *M. bovis* occurs four times more often, being the third most frequent bacterial coinfection and accounting for 14.81% (4/27) of all bacterial coinfections. Similar to the results obtained by us and Paller et al., a significant proportion of coinfections were configurations containing *P. multocida*. They showed that in their study, BRSV was the most frequently reported coinfection along with *P. multocida* (58.21% coinfection) [32]. In our study, this configuration appears only twice, accounting for only 2.82% (2/71) of all results. Pratelli et al. showed in their study that the most common coinfection recorded was that containing BCoV and *H. somni* [8]. Although our study did not record the occurrence of such a configuration, we did show a statistically significant *phi contingency* coefficient for these pathogens. The most common configuration of bacterial-virial coinfection was: BCoV, *P. multocida*, and *H. somni;* however, it appeared 3 times only.

### 4.4. Correlations

To date, much of the literature has focused on demonstrating the correlation of environmental factors to the occurrence of BRD symptoms, such as stress associated with calf transport [8,10]. Less is known about the correlation between individual infectious factors in the course of BRD in calves. Statistical analysis revealed five statistically significant correlations between pathogens with three with an average-level strength of effect and two with a low level of effect. The strongest correlation is between *H. somni* and *M. bovis* bacteria. This correlation was also shown by Andrés-Lasheras et al. [2]. The next one shown between bacteria also occurred with *M. bovis*, but it had the lowest strength of effect of a given association and it was the correlation with *M. haemolytica*. The highest strength of effect of a correlation between viral agents was shown between BRSV and BPIV-3 and was at an average level. On the other hand, although correlations were shown between BRSV and BPIV-3, their prevalence in our study was low. All statistically significant correlations showed a positive association. In other words, the more frequently one pathogen was recorded, the higher the chance of detecting the other pathogen from a given association. Nonetheless, the correlation between *M. haemolytica* and BRSV was inverse. What it means is that *M. haemolytica* was significantly less frequent with concurrent BRSV, but this relationship is at the limit of statistical significance, with a low strength of effect. A study of Canadian cattle by Andres-Lasheras et al. showed a correlation in cattle between *M. haemolytica* and *H. somni* and between *P. multocida* and *M. bovis* [2]. In our study, correlations between these pathogens were not detected. There are no statistically significant correlations between *P. multocida* and other pathogens.

### 4.5. Coexistence

The complexity of the relationships between pathogens and the very high prevalence of *P. multocida* make it difficult to select a single ideal statistical test that demonstrates these relationships. Therefore, the relationships between pathogens were examined not only by correlations, but also by the frequency of occurrence together, which are represented graphically using sets in this study (Figure 6 and Figure 7) in order to better show relationships between tested pathogens through coexistence in coinfections.

The Jaccard index tells what percentage of all elements in the two sets they share. Saegerman et al. showed this index for the same pathogens that we detected. The highest Jaccard indexes in their study and in ours are those for associations between bacteria [20]. In their study, as in ours, the high Jaccard index was in the set of *P. multocida* and *M. haemolytica*, although the highest they recorded was for *P. multocida* and *M. bovis*. Similarly to correlations, the high prevalence of *P. multocida* makes it difficult to interpret the results. Nevertheless, the Jaccard index in the Saegerman et al. study suggests that *P. multocida* was also present along with *M. haemolytica* in the cattle population they studied. For the *M. bovis* and *H. somni* set also, they showed a high Jaccard index, as did we [20]. Considering also the correlation results presented earlier with the highest phi Yule contingency coefficient for this association and the correlations shown by Andrés-Lasheras et al., this suggests that there is a relationship/dependence between *M. bovis* and *H. somni* [2]. In the Saegerman et al. study, the Jaccard indexes were low for sets consisting of the viruses we studied [20]. In our study, they are higher; nevertheless, the number of occurrences of these pathogens was low, so the index we obtained may be less reliable than that obtained by Saegerman et al. [20]. The Jaccard index tells us the percentage for both sets; it does not show whether either set was more involved in the formation of the common part. Therefore, we also analyzed for chosen pathogens what proportion of a single set is the common part, which we showed by sets (Figure 6 and Figure 7). It was noted that BCoV was more frequently recorded with *M. haemolytica* than the other way around. The highest disproportion of the level of coexistence in coinfections was between BPIV-3 and BCoV. BPIV-3 more frequently coexisted with BCoV than the other way around. The coexistence level of other tested pathogen pairs that show correlations was more proportional and did not exceed ten percentage points.

Taking into consideration the shown bacterial agents, prevalence, and scientific reports about opportunistic pathogens, it is important to bear in mind that despite the very high prevalence of *P. multocida* and the high frequency of coinfection with *P. multocida* and *M. haemolytica* in some of the calves we examined, these agents were not necessarily responsible for the presence of disease symptoms [8,17,28,51,52]. Nevertheless, being components of the respiratory tract flora, when the clinical form of the disease occurs (regardless of whether they are the cause) due to the use of antimicrobials in the treatment and control of BRD, they acquire resistance to the antibiotics used, becoming a reservoir of resistance genes [2,14,36,38,52,53,54]. It is also important to perform drug resistance testing with determination of the presence of chemotherapeutic resistance genes in *P. multocida* and *M. haemolytica* isolated from dairy cattle calves from Poland. Moreover, we believe that the subject of relationships between pathogens in the course of BRD requires further studies.

## Figures and Tables

**Table 1:** The table shows the correlations between the occurrence of the pathogen pairs studied. Statistically significant correlations and their strength of effect are highlighted in bold. All marked correlations were positive. *p* denotes statistical significance; ϕ denotes a Phi contingency coefficient indicating the strength of effect of a given association. * denotes that the correlation shown is inverse.

Correlations							
		BRSV	BCoV	*Mannheimia haemolytica*	*Pasteurella multocida*	*Histophilus somni*	*Mycoplasma bovis*
BPIV-3	*p*	**0.010**	**0.011**	1.000	1.000	1.000	0.713
	ϕ	**0.33**	**0.32**	0.04	0.03	0.01	0.08
BRSV	*p*		0.103	0.068 *	0.584	0.680	0.688
	ϕ		0.22	0.23 *	0.130	0.070	0.06
BCoV	*p*			0.726	1.000	**0.021**	0.311
	ϕ			0.04	0.01	**0.27**	0.12
*Mannheimia haemolytica*	*p*				1.000	1.000	**0.032**
	ϕ				<0.01	0.02	**0.25**
*Pasteurella multocida*	*p*					1.000	0.265
	ϕ					0.05	0.15
*Histophilus somni*	*p*						**0.001**
	ϕ						**0.38**

**Table 2:** Table showing configurations of pathogens detected, with percentages of occurrence. NS—nasal swabs, TW—tracheal washes.

Configurations					
No. of Pathogen in Configura-tions	Pathogens Name	No. of Positive Results			% of All Positive Results
		All (71)	NS (45)	TW (26)	
1	BCoV	1	1	0	1.41%
	*M. haemolytica*	2	2	0	2.82%
	*P. multocida*	13	9	4	18.31%
2	*M. haemolytica*, BPIV-3	1	1	0	1.41%
	*P. multocida*, BPIV-3	1	1	0	1.41%
	*P. multocida*, BRSV	2	1	1	2.82%
	*P. multocida*, BCoV	3	3	0	4.23%
	*P. multocida*, *M. haemolytica*	10	7	3	14.08%
	*P. multocida*, *H. somni*	3	2	1	4.23%
	*P. multocida*, *M. bovis*	2	0	2	2.82%
3	BPIV-3, BCoV, *P. multocida*	1	1	0	1.41%
	BPIV-3, BRSV, *P. multocida*	1	1	0	1.41%
	BCoV, *H. somni*, *M. bovis*	1	0	1	1.41%
	BCoV, *P. multocida*, *H. somni*	3	3	0	4.23%
	BCoV, *P. multocida*, *M. haemolytica*	2	1	1	2.82%
	BCoV, *M. haemolytica*, *H. somni*	1	0	1	1.41%
	*M. haemolytica*, *P. multocida*, *M. bovis*	5	3	2	7.04%
	*M. haemolytica*, *P. multocida.*, *H. somni*	1	1	0	1.41%
	*P. multocida*, *H. somni*, *M. bovis*	2	0	2	2.82%
4	BPIV-3, BRSV, BCoV, *P. multocida*	1	1	0	1.41%
	BPIV-3, BCoV, *P. multocida*, *M. haemolytica*	2	1	1	2.82%
	BRSV, BCoV, *P. multocida*, *M. bovis*	1	0	1	1.41%
	BCoV, *P. multocida*, *M. haemolytica*, *M. bovis*	2	1	1	2.82%
	*P. multocida*, *M. haemolytica*, *H. somni*, *M. bovis*	4	1	3	5.63%
5	BPIV-3, BRSV, BCoV, *P. multocida*, *H. somni*	1	1	0	1.41%
	BPIV-3, BCoV, *P. multocida*, *H. somni*, *M. bovis*	1	1	0	1.41%
	BRSV, BCoV, *P. multocida*, *H. somni*, *M. bovis*	1	1	0	1.41%
	BCoV, *P. multocida*, *H. somni*, *M. haemolytica*, *M. bovis*	2	1	1	2.82%
6	not detected	-	-	-	-
7	BPIV-3, BRSV, BCoV, *P. multocida*, *M. haemolytica*, *H. somni*, *M. bovis*	1	0	1	1.41%

## Data Availability

Data available on request from the corresponding author.

## References

[1] Witkowska D., Ponieważ A. (2022). The Effect of Housing System on Disease Prevalence and Productive Lifespan of Dairy Herds—A Case Study. Animals.

[2] Andrés-Lasheras S., Ha R., Zaheer R., Lee C., Booker C.W., Dorin C., van Donkersgoed J., Deardon R., Gow S., Hannon S.J. (2021). Prevalence and Risk Factors Associated with Antimicrobial Resistance in Bacteria Related to Bovine Respiratory Disease-A Broad Cross-Sectional Study of Beef Cattle at Entry into Canadian Feedlots. Front. Vet. Sci..

[3] Van der Poel W.H.M., Brand A., Kramps J.A., van Oirschot J.T. (1994). Respiratory syncytial virus infections in human beings and in cattle. J. Infect..

[4] Dubrovsky S.A., van Eenennaam A.L., Karle B.M., Rossitto P.V., Lehenbauer T.W., Aly S.S. (2019). Bovine respiratory disease (BRD) cause-specific and overall mortality in preweaned calves on California dairies: The BRD 10K study. J. Dairy Sci..

[5] Murray G.M., More S.J., Sammin D., Casey M.J., McElroy M.C., O’Neill R.G., Byrne W.J., Earley B., Clegg T.A., Ball H. (2017). Pathogens, patterns of pneumonia, and epidemiologic risk factors associated with respiratory disease in recently weaned cattle in Ireland. J. Vet. Diagn. Investig..

[6] O’Neill R., Mooney J., Connaghan E., Furphy C., Graham D.A. (2014). Patterns of detection of respiratory viruses in nasal swabs from calves in Ireland: A retrospective study. Vet. Rec..

[7] Studer E., Schönecker L., Meylan M., Stucki D., Dijkman R., Holwerda M., Glaus A., Becker J. (2021). Prevalence of BRD-Related Viral Pathogens in the Upper Respiratory Tract of Swiss Veal Calves. Animals.

[8] Pratelli A., Cirone F., Capozza P., Trotta A., Corrente M., Balestrieri A., Buonavoglia C. (2021). Bovine respiratory disease in beef calves supported long transport stress: An epidemiological study and strategies for control and prevention. Res. Vet. Sci..

[9] Pratelli A., Padalino B. (2022). Editorial: Evolving Prospects of Bovine Respiratory Diseases and Management in Feedlot Cattle. Front. Vet. Sci..

[10] Taylor J.D., Fulton R.W., Lehenbauer T.W., Step D.L., Confer A.W. (2010). The epidemiology of bovine respiratory disease: What is the evidence for predisposing factors?. Can. Vet. J. Rev. Vet. Can..

[11] Nickell J.S., White B.J. (2010). Metaphylactic antimicrobial therapy for bovine respiratory disease in stocker and feedlot cattle. Vet. Clin. N. Am. Food Anim. Pract..

[12] Confer A.W. (2009). Update on bacterial pathogenesis in BRD. Anim. Health Res. Rev..

[13] Guterbock W.M. (2014). The impact of BRD: The current dairy experience. Anim. Health Res. Rev..

[14] Akalu M., Vemulapati B., Abayneh T., Degefa T., Deresse G., Gelaye E. (2022). Serotyping, antibiogram, and detection of bacterial pathogens associated with bovine respiratory disease in selected areas of Ethiopia. Ir. Vet. J..

[15] Fanelli A., Cirilli M., Lucente M.S., Zarea A.A.K., Buonavoglia D., Tempesta M., Greco G. (2021). Fatal Calf Pneumonia Outbreaks in Italian Dairy Herds Involving Mycoplasma bovis and Other Agents of BRD Complex. Front. Vet. Sci..

[16] Hashem Y.M., Mousa W.S., Abdeen E.E., Abdelkhalek H.M., Nooruzzaman M., El-Askary A., Ismail K.A., Megahed A.M., Abdeen A., Soliman E.A. (2022). Prevalence and Molecular Characterization of Mycoplasma Species, Pasteurella multocida, and Staphylococcus aureus Isolated from Calves with Respiratory Manifestations. Animals.

[17] Sarchet J.J., Pollreisz J.P., Bechtol D.T., Blanding M.R., Saltman R.L., Taube P.C. (2022). Limitations of bacterial culture, viral PCR, and tulathromycin susceptibility from upper respiratory tract samples in predicting clinical outcome of tulathromycin control or treatment of bovine respiratory disease in high-risk feeder heifers. PLoS ONE.

[18] Kishimoto M., Tsuchiaka S., Rahpaya S.S., Hasebe A., Otsu K., Sugimura S., Kobayashi S., Komatsu N., Nagai M., Omatsu T. (2017). Development of a one-run real-time PCR detection system for pathogens associated with bovine respiratory disease complex. J. Vet. Med. Sci..

[19] Pardon B., de Bleecker K., Dewulf J., Callens J., Boyen F., Catry B., Deprez P. (2011). Prevalence of respiratory pathogens in diseased, non-vaccinated, routinely medicated veal calves. Vet. Rec..

[20] Saegerman C., Gaudino M., Savard C., Broes A., Ariel O., Meyer G., Ducatez M.F. (2022). Influenza D virus in respiratory disease in Canadian, province of Québec, cattle: Relative importance and evidence of new reassortment between different clades. Transbound. Emerg. Dis..

[21] Mekata H., Yamamoto M., Hamabe S., Tanaka H., Omatsu T., Mizutani T., Hause B.M., Okabayashi T. (2018). Molecular epidemiological survey and phylogenetic analysis of bovine influenza D virus in Japan. Transbound. Emerg. Dis..

[22] Van der Fels-Klerx H.J., Saatkamp H.W., Verhoeff J., Dijkhuizen A.A. (2002). Effects of bovine respiratory disease on the productivity of dairy heifers quantified by experts. Livest. Prod. Sci..

[23] Lowie T., van Leenen K., Jourquin S., Pas M.L., Bokma J., Pardon B. (2022). Differences in the association of cough and other clinical signs with ultrasonographic lung consolidation in dairy, veal, and beef calves. J. Dairy Sci..

[24] Sacco R.E., McGill J.L., Pillatzki A.E., Palmer M.V., Ackermann M.R. (2014). Respiratory syncytial virus infection in cattle. Vet. Pathol..

[25] Closs G., Dechow C. (2017). The effect of calf-hood pneumonia on heifer survival and subsequent performance. Livest. Sci..

[26] Wang M., Schneider L.G., Hubbard K.J., Smith D.R. (2018). Cost of bovine respiratory disease in preweaned calves on US beef cow-calf operations (2011–2015). J. Am. Vet. Med. Assoc..

[27] Chai J., Capik S.F., Kegley B., Richeson J.T., Powell J.G., Zhao J. (2022). Bovine respiratory microbiota of feedlot cattle and its association with disease. Vet. Res..

[28] Centeno-Martinez R.E., Glidden N., Mohan S., Davidson J.L., Fernández-Juricic E., Boerman J.P., Schoonmaker J., Pillai D., Koziol J., Ault A. (2022). Identification of bovine respiratory disease through the nasal microbiome. Anim. Microbiome.

[29] Timsit E., Workentine M., van der Meer F., Alexander T. (2018). Distinct bacterial metacommunities inhabit the upper and lower respiratory tracts of healthy feedlot cattle and those diagnosed with bronchopneumonia. Vet. Microbiol..

[30] Winnicki S., Jugowar L.J. (2013). Housing systems for dairy cattle in the Wielkopolska region–Current status and prospects. Przegląd Hod..

[31] Thrusfield M., Brown H., Christley R. (2018). Veterinary Epidemiology, 4th ed.

[32] Paller T., Hostnik P., Pogačnik M., Toplak I. (2017). The prevalence of ten pathogens detected by a real-time pcr method in nasal swab samples collected from live cattle with respiratory disease. Slov. Vet. Res..

[33] Oliveira T.E.S., Scuisato G.S., Pelaquim I.F., Cunha C.W., Cunha L.S., Flores E.F., Pretto-Giordano L.G., Lisbôa J.A.N., Alfieri A.A., Saut J.P.E. (2021). The Participation of a Malignant Catarrhal Fever Virus and Mycoplasma bovis in the Development of Single and Mixed Infections in Beef and Dairy Cattle with Bovine Respiratory Disease. Front. Vet. Sci..

[34] Pardon B., Buczinski S. (2020). Bovine Respiratory Disease Diagnosis: What Progress Has Been Made in Infectious Diagnosis?. Vet. Clin. N. Am. Food Anim. Pract..

[35] Klima C.L., Zaheer R., Cook S.R., Booker C.W., Hendrick S., Alexander T.W., McAllister T.A. (2014). Pathogens of bovine respiratory disease in North American feedlots conferring multidrug resistance via integrative conjugative elements. J. Clin. Microbiol..

[36] Jamali H., Rezagholipour M., Fallah S., Dadrasnia A., Chelliah S., Velappan R.D., Wei K.S.C., Ismail S. (2014). Prevalence, characterization and antibiotic resistance of Pasteurella multocida isolated from bovine respiratory infection. Vet. J..

[37] Schönecker L., Schnyder P., Schüpbach-Regula G., Meylan M., Overesch G. (2020). Prevalence and antimicrobial resistance of opportunistic pathogens associated with bovine respiratory disease isolated from nasopharyngeal swabs of veal calves in Switzerland. Prev. Vet. Med..

[38] Timsit E., Hallewell J., Booker C., Tison N., Amat S., Alexander T.W. (2017). Prevalence and antimicrobial susceptibility of Mannheimia haemolytica, Pasteurella multocida, and Histophilus somni isolated from the lower respiratory tract of healthy feedlot cattle and those diagnosed with bovine respiratory disease. Vet. Microbiol..

[39] Headley S.A., Okano W., Balbo L.C., Marcasso R.A., Oliveira T.E., Alfieri A.F., Negri Filho L.C., Michelazzo M.Z., Rodrigues S.C., Baptista A.L. (2018). Molecular survey of infectious agents associated with bovine respiratory disease in a beef cattle feedlot in southern Brazil. J. Vet. Diagn. Investig..

[40] Hodnik J.J., Ježek J., Starič J. (2020). Coronaviruses in cattle. Trop. Anim. Health Prod..

[41] Van Leenen K., van Driessche L., de Cremer L., Masmeijer C., Boyen F., Deprez P., Pardon B. (2020). Comparison of bronchoalveolar lavage fluid bacteriology and cytology in calves classified based on combined clinical scoring and lung ultrasonography. Prev. Vet. Med..

[42] Frucchi A.P.S., Dall Agnol A.M., Bronkhorst D.E., Beuttemmuller E.A., Alfieri A.A., Alfieri A.F. (2022). Bovine Coronavirus Co-infection and Molecular Characterization in Dairy Calves with or Without Clinical Respiratory Disease. Front. Vet. Sci..

[43] Urban-Chmiel R., Wernicki A., Puchalski A., Dec M., Stęgierska D., Grooms D.L., Barbu N.I. (2015). Detection of bovine respiratory syncytial virus infections in young dairy and beef cattle in Poland. Vet. Q..

[44] Szacawa E., Niemczuk K., Dudek K., Bednarek D., Rosales R., Ayling R. (2015). Mycoplasma bovis infections and co-infections with other Mycoplasma spp. with different clinical manifestations in affected cattle herds in eastern region of Poland. Bull. Vet. Inst. Pulawy.

[45] Doyle D., Credille B., Lehenbauer T.W., Berghaus R., Aly S.S., Champagne J., Blanchard P., Crossley B., Berghaus L., Cochran S. (2017). Agreement among 4 Sampling Methods to Identify Respiratory Pathogens in Dairy Calves with Acute Bovine Respiratory Disease. J. Vet. Intern. Med..

[46] Zhang M., Hill J.E., Fernando C., Alexander T.W., Timsit E., van der Meer F., Huang Y. (2019). Respiratory viruses identified in western Canadian beef cattle by metagenomic sequencing and their association with bovine respiratory disease. Transbound. Emerg. Dis..

[47] Zhang M., Hill J.E., Alexander T.W., Huang Y. (2021). The nasal viromes of cattle on arrival at western Canadian feedlots and their relationship to development of bovine respiratory disease. Transbound. Emerg. Dis..

[48] McMullen C., Alexander T.W., Orsel K., Timsit E. (2020). Progression of nasopharyngeal and tracheal bacterial microbiotas of feedlot cattle during development of bovine respiratory disease. Vet. Microbiol..

[49] Thomas A., Dizier I., Trolin A., Mainil J., Linden A. (2002). Comparison of sampling procedures for isolating pulmonary mycoplasmas in cattle. Vet. Res. Commun..

[50] Van Driessche L., Valgaeren B.R., Gille L., Boyen F., Ducatelle R., Haesebrouck F., Deprez P., Pardon B. (2017). A Deep Nasopharyngeal Swab Versus Nonendoscopic Bronchoalveolar Lavage for Isolation of Bacterial Pathogens from Preweaned Calves with Respiratory Disease. J. Vet. Intern. Med..

[51] Bassel L.L., Kaufman E.I., Alsop S.-N.A., Buchan J., Hewson J., McCandless E.E., Tiwari R., Sharif S., Vulikh K., Caswell J.L. (2021). Effect of aerosolized bacterial lysate on development of naturally occurring respiratory disease in beef calves. J. Vet. Intern. Med..

[52] Kudirkiene E., Aagaard A.K., Schmidt L.M.B., Pansri P., Krogh K.M., Olsen J.E. (2021). Occurrence of major and minor pathogens in calves diagnosed with bovine respiratory disease. Vet. Microbiol..

[53] Klima C.L., Holman D.B., Ralston B.J., Stanford K., Zaheer R., Alexander T.W., McAllister T.A. (2019). Lower Respiratory Tract Microbiome and Resistome of Bovine Respiratory Disease Mortalities. Microb. Ecol..

[54] Zeineldin M., A Elolimy A., Barakat R. (2020). Meta-analysis of bovine respiratory microbiota: Link between respiratory microbiota and bovine respiratory health. FEMS Microbiol. Ecol..

